# Supplementary material for: Heterogeneous Formation of DNA Double-Strand Breaks and Cell-Free DNA in Leukemia T-Cell Line and Human Peripheral Blood Mononuclear Cells in Response to Topoisomerase II Inhibitors
Source: Cancers (Basel). 2024 Nov 12;16(22):3798. doi: 10.3390/cancers16223798 (PMC11592837; doi:10.3390/cancers16223798)
Supplement: Supplementary file 1 [file cancers-16-03798-s001.zip › cancers-3276659-supplementary.pdf]

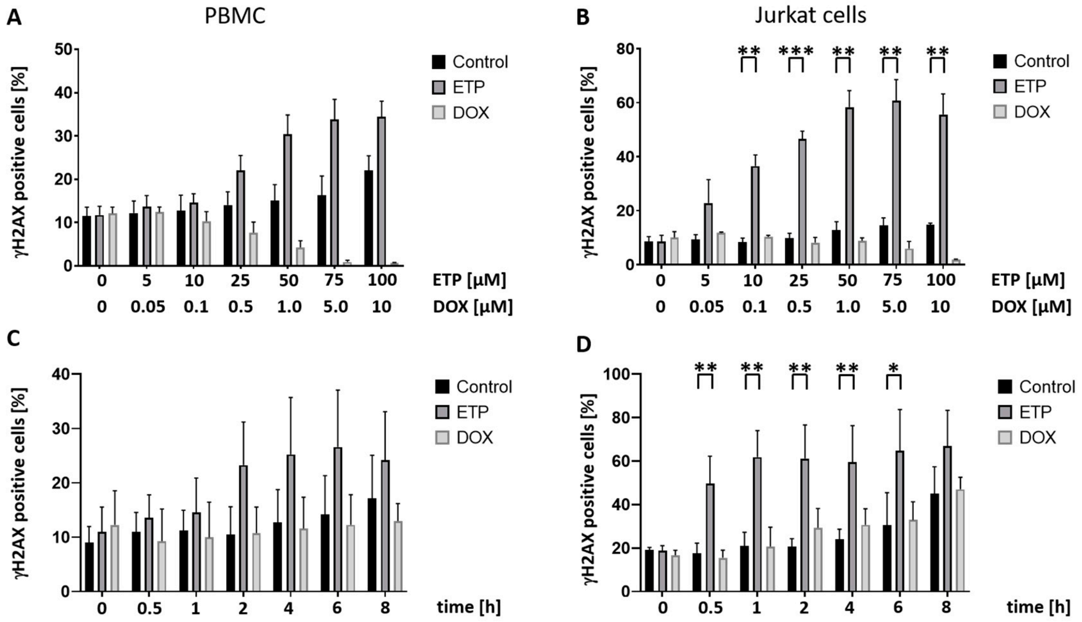

Supplemental Figure S1. Dose- and time-dependent studies on γH2AX-stained PBMCs and Jurkat cells in response to ETP or DOX: FACS analysis on dose-dependent γH2AX formation in (**A**) PBMCs and (**B**) Jurkat cells treated with increasing concentrations of ETP and DOX for 3 h. DMSO-treated cells served as the control. FACS analysis on time-dependent γH2AX formation in (**C**) PBMCs and (**D**) Jurkat cells between 0 and 8 h of incubation with either ETP (25 µM) or DOX (1.0 µM). Bars represent the mean and standard deviation of three independent experiments in dose- and four independent experiments in time course analyses. Numbers of asterisks indicate increasing significance levels: * $p \leq 0.05$, ** $p \leq 0.01$, *** $p \leq 0.001$. ETP: etoposide; DOX: doxorubicin.

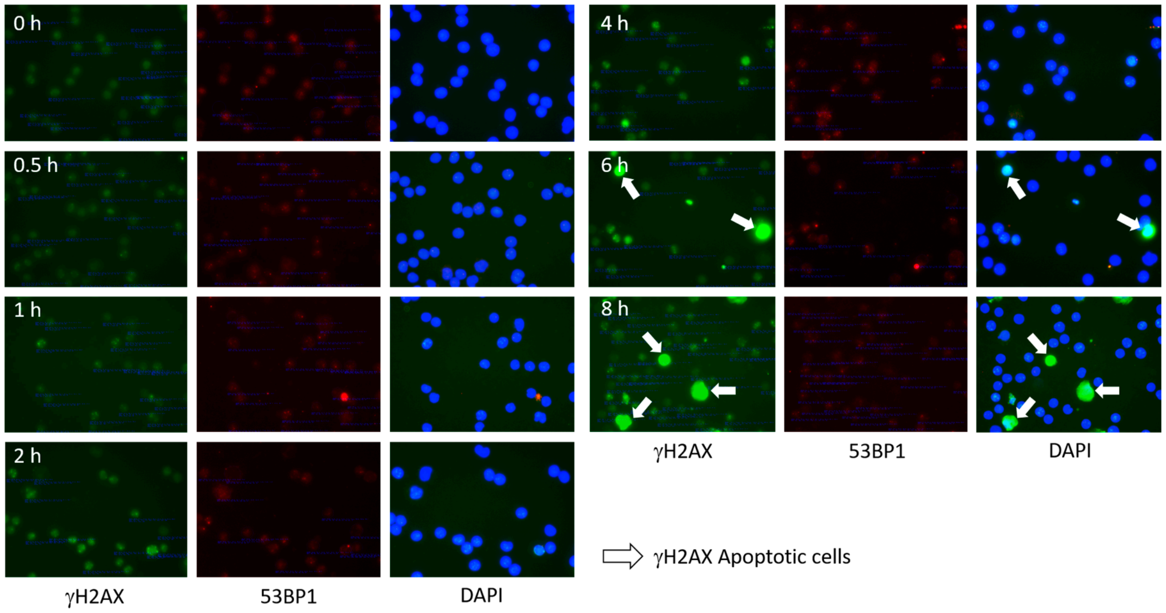

Supplemental Figure S2. Time-dependent γH2AX staining associated with cell death in ETP-treated PBMCs: Representative images taken by the AKLIDES platform after immunofluorescent staining of cells treated with 25 µM ETP between 0 and 8 h. Nuclear pan-stained γH2AX (green) is indicated by white arrows. As control, 53BP1-specific staining (red) is shown. Respective images

of DAPI-stained nuclei (blue) are merged with γH2AX- and 53BP1-specific signals. ETP: etoposide.

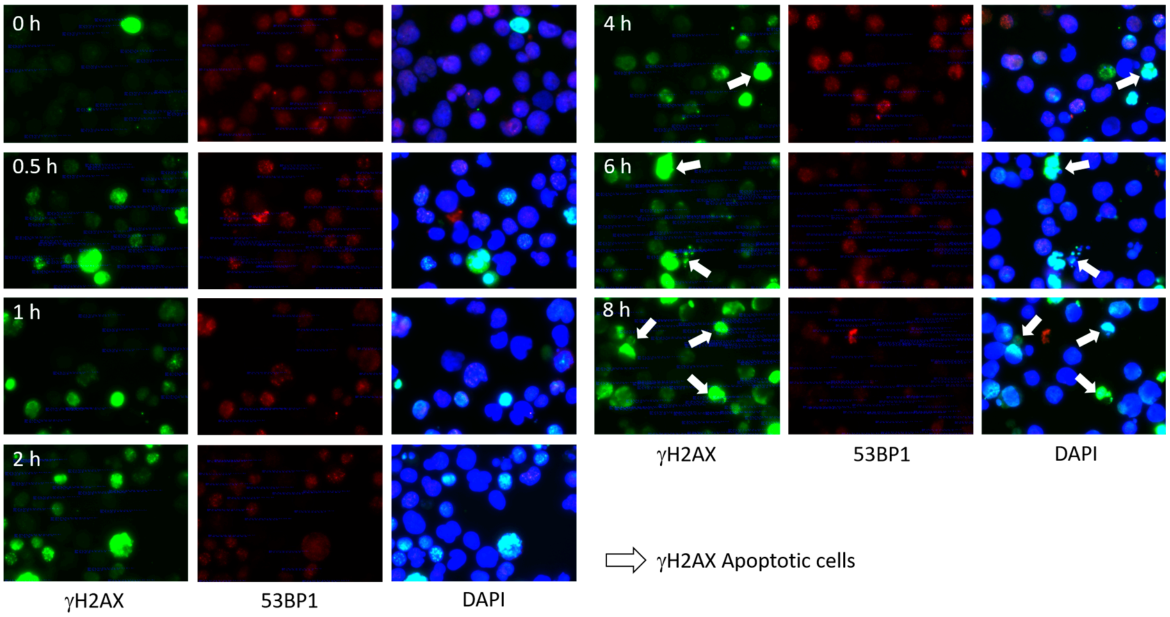

Supplemental Figure S3. Time-dependent γH2AX staining associated with cell death in ETP-treated Jurkat cells: Representative images taken by the AKLIDES platform after immunofluorescent staining of cells treated with 25 µM ETP between 0 and 8 h. Nuclear pan-stained γH2AX (green) is indicated by white arrows. As control, 53BP1-specific staining (red) is shown. Respective images of DAPI-stained nuclei (blue) are merged with γH2AX- and 53BP1-specific signals. ETP: etoposide.

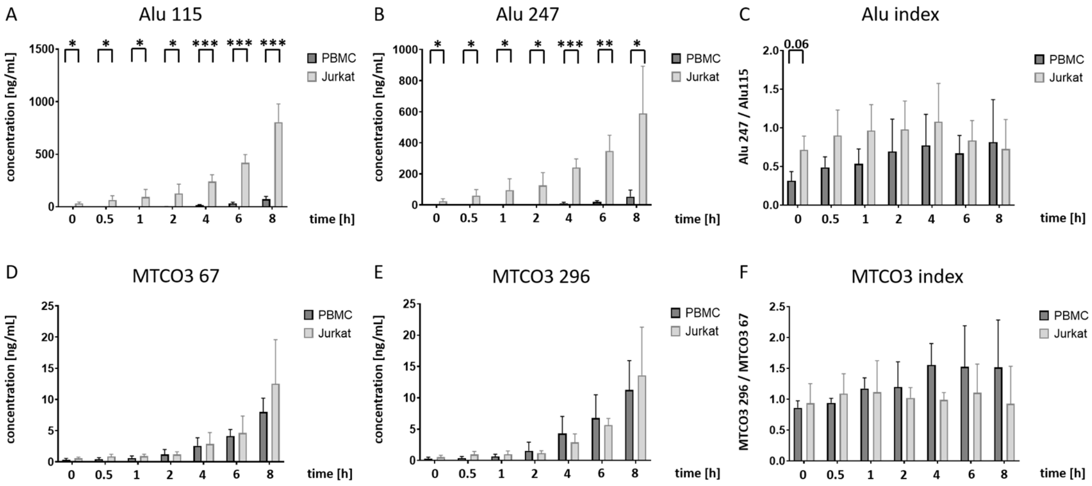

Supplemental Figure S4. Time-dependent quantification of nuclear and mitochondrial cfDNA in PBMCs and Jurkat cells. CfDNA fragment concentrations after qPCR analysis using supernatant medium at the indicated time points (0–8 h) as a template. Nuclear cfDNA concentrations (in ng/mL) of (A) Alu 115 bp and (B) Alu 247 bp as well as (C) the corresponding DII values (dimensionless) in PBMCs compared to Jurkat cells. Mitochondrial cfDNA concentrations (in ng/ml) of (D) MTCO3 67 bp and (E) MTCO3 296 bp as well as (F) the corresponding DII values of PBMCs compared to Jurkat cells. Data represent the mean and standard deviation of four independent experiments. Numbers of asterisks indicate increasing significance levels: * $p \leq 0.05$, ** $p \leq 0.01$, *** $p \leq 0.001$.

List of abbreviations:

DSB	DNA double-strand breaks
cfDNA	Circulating cell-free DNA
n-cfDNA	Nuclear cell-free DNA
mt-cfDNA	Mitochondrial cell-free DNA
MTCO3	Mitochondrially encoded cytochrome c oxidase III
γH2AX	Gamma-Histon 2AX
DDR	DNA damage response
qPCR	Quantitative real-time polymerase chain reaction
DOX	Doxorubicin
ETP	Etoposide
DMSO	Dimethyl sulfoxide
PBMC	Peripheral blood mononuclear cells
ATM	Ataxia-telangiectasia mutated
NHJ	Non-homologous end joining
HR	Homologues recombination
FACS	Fluorescence-activated cell sorting
53BP1	p53 binding protein 1
BRCA1	Breast cancer gene protein 1
PUMA	p53 upregulated modulator of apoptosis
Bax	Bcl-2-associated X protein
TP53	Tumor protein p53
DII	DNA integrity index
ALL	Acute lymphoblastic leukemia
DAPI	4',6-Diamidino-2-phenylindol
FCS	Fetal calf serum
